# Salvianolic acid B reduced the formation of epidural fibrosis in an experimental rat model

**DOI:** 10.1186/s13018-016-0475-x

**Published:** 2016-11-16

**Authors:** Feng Chen, Changyao Wang, Jintang Sun, Jin Wang, Lanfeng Wang, Jianmin Li

**Affiliations:** 1 Qilu Hospital, Shandong University, Wenhua Xi Road 107th, Jinan, People’s Republic of China; 2 Department of Trauma, The Affiliated Hospital of Qingdao University, Qingdao, People’s Republic of China; 3 Department of Joint Surgery, The Affiliated Hospital of Qingdao University, Qingdao, People’s Republic of China; 4 Department of Joint Surgery, The People’s Hospital of Jimo City, Qingdao, People’s Republic of China

**Keywords:** Animal experimentation, Laminectomy, Salvia

## Abstract

**Background:**

Salvianolic acid B (Sal B) was newly reported to be able to attenuate fibrosis in the animal model. The aim of the present study was to investigate the effect of the intragastric application of Sal B on the prevention of epidural fibrosis (EF).

**Methods:**

Forty healthy adult male Wistar rats were divided into four treatment groups (*n* = 10 per group): (1) 10 mg/kg Sal B, (2) 30 mg/kg Sal B, (3) 50 mg/kg Sal B and (4) Saline (vehicle treatment, control group). All animals underwent a laminectomy at the lumbar 1–2 (L 1–2) level. After intragastric treatment, all rats were sacrificed at post-operative week 8. The extent of the epidural scar, the regeneration of the vasculature and the expression levels of vascular endothelial growth factor (VEGF) were analysed.

**Results:**

The animals’ recovery was uneventful during the experimental period. The extent of the epidural scar, the regeneration of the vasculature and the expression levels of VEGF suggested better outcomes in the Sal B-treated groups. Sal B exerted the ability to prevent the formation of an epidural scar and vascularization at the laminectomy sites. The effects of Sal B were dose-dependent, with the 50 mg/kg Sal B group showing the best outcomes compared with the other groups.

**Conclusions:**

Post-operative intragastric application of Sal B can prevent the formation of epidural scarring. Sal B exerted these effects in a dose-dependent manner, and 50 mg/kg dose was shown to be the best effect in the present study. The results of this study reveal that Sal B could be a potential therapy for EF and valuable for further research.

## Background

Epidural fibrosis (EF) is widely accepted as one of the most common factors that contribute to failed back surgery syndrome (FBSS) [1]. FBSS is defined as continuous low back pain with or without radicular pain after lumbar surgery. It was estimated that 25% of post-operative lumbar patients could suffer FBSS due to EF [2]. EF is defined as the excessive formation of scar tissue in the epidural space following lumbar laminectomy [3]. As previously reported, EF is caused by multiple factors including nerve root tethering, interference of cerebrospinal fluid flow, destruction of nerve root vascular supply and dural compression [4].

Considering that another surgery at the same site to excise the epidural scar could also lead the recurrence of the scar [5], the current widely accepted modalities for overcoming EF is to either inhibit or prevent the formation of excessive scar tissue [6, 7]. Many attempts have been conducted at both the clinical and experimental levels [8–10]. However, none of these efforts have resulted in successful clinical application.

Salvianolic acid B (Sal B) is one of the major water-soluble bioactive component of the traditional Chinese medical agent, *Salvia miltiorrhiza*, and has been implemented as a treatment for cardiovascular diseases [11, 12]. In our previous study, we evaluated the intragastric application of Sal B on the prevention of EF in vivo [13]. Based on our results and the latest studies [13, 14], it was revealed that Sal B is able to exert anti-fibrotic effect through inhibition of the transforming growth factor-β (TGF-β) signaling pathway. At the same time, another studies looking into Sal B’s anti-inflammatory and anti-fibrotic effects in different rat models were also recently reported [15–17]. Thus, Sal B was thought to be potentially beneficial for attenuating inflammatory and fibrotic process. In our previous study with a laminectomy rat model, only one dose of 30 mg/kg Sal B was used. In order to further evaluate the effect of Sal B in a long-term setting and define the most effective dose and determine the high-dose toxicity, the present study was performed.

## Methods

### Animals

Forty healthy adult male Wistar rats (weight 250 ± 20 g) were used. The present experiment was approved by the University Laboratory Animal Care Committee and was performed in compliance with the European Communities Council Directive (86/809/EEC) and with the principles of International Laboratory Animal Care. Animals were pre-operatively housed in the Qingdao University animal laboratory for 10 days. The animals randomly divided into four groups based on the different intragastric treatments (10 rats per group): (1) 10 mg/kg Sal B treatment, (2) 30 mg/kg Sal B treatment, (3) 50 mg/kg Sal B treatment, and (4) Saline group (vehicle treatment, as control group). All the rats were euthanized with an anaesthesia overdose at post-operative 8 weeks.

### Salvianolic acid B administration

Sal B treatment groups: salvianolic acid B (10, 30, and 50 mg/kg diluted in saline) or vehicle (saline) was administered intragastrically [13, 17]. Vehicle treatment group: The same volume of saline was given in the same way. Varying concentrations of Sal B or vehicle (saline) was daily administered intragastrically from post-operative 1 day to post-operative 56 days.

### Implementation of the laminectomy rat model

The laminectomy rat model was produced as previously reported [6, 13]. Generally, after general anaesthesia with 10% chloral hydrate (0.3 ml/100 g body weight), all of the rats were restrained on a temperature pad (setting temperature 30 °C) in the prone position. All of the rats were individually marked with ear tags. The rats’ lower back was shaved and sterilized with a 10% polyvinylpyrrolidone/iodine solution. A midline incision was made from lumbar 1 to lumbar 2 (L1 to L2). After removing the paraspinal musculature, the L1–L2 laminectomy was performed. Attention was paid to avoid traumatizing the dura and the nerve roots. After adequate haemostasis, the incision was surgically closed in layers.

### EF macroscopic evaluation

Eight weeks after the surgery, a macroscopic evaluation was conducted. All groups were evaluated based on the Rydell classification under the double-blind principle (Table 1) [7, 10]. Generally, while the rats were under general anaesthesia, the surgical incision was carefully reopened. The state of the epidural scar adhesion was evaluated with the help of trained assistants.

**Table 1:** Rydell classification

Grade 0	Epidural scar tissue was not adherent to the dura mater.
Grade 1	Epidural scar tissue was adherent to the dura mater, but easily dissected.
Grade 2	Epidural scar tissue was adherent to the dura mater and difficultly dissected without disrupting the dura matter.
Grade 3	Epidural scar tissue was firmly adherent to the dura mater and could not be dissected.

### Histological analysis

At post-operative week 8, a histological analysis was performed. Five rats from each group were randomly selected and euthanized. The entire L1 vertebral column, including the muscle and scar tissue, was collected and fixed in a 10% phosphate-buffered formaldehyde solution. After dehydration and decalcification with a Cal-Ex II solution, the samples were placed in paraffin, and 5-μm axial sections of the surgery site were made. The sections were subjected to immunohistochemistry and stained with haematoxylin and eosin (H&E). The epidural adhesion status and any vessel regeneration were detected via H&E staining under a light microscope. To quantify the fibroblast numbers, immunohistochemistry with a vimentin antibody (AB92547, 1:400, Abcam, USA) was performed, and the intensity was calculated.

### Western blot

As previously reported, the expressional level of vascular endothelial growth factor (VEGF) was decreased after treatment with Sal B [13]. To determine whether the Sal B-induced changes in the VEGF levels were dose-dependent, Western blot targeting VEGF was performed at post-operative week 8 in the present study. Generally, five rats from each group were randomly selected and euthanized. The epidural scars were collected from the surgical sites. Then, RIPA lysis buffer was added to lyse and homogenize the samples. Equivalent protein amounts (80 μg total protein) were pre-stained with a marker (#SM0671, Thermo Fisher Scientific) in a buffer containing 192 mM glycine, 0.1% sodium dodecyl sulfate (SDS) and 24.8 mM Tris. SDS-polyacrylamide gels (10%) were run at 80 V for 30 min and 120 V for 2 h to resolve the proteins. After the precipitated proteins were separated, they were transferred to PVDF membranes (LC2005, Thermo Fisher Scientific) using the following transfer buffer: 10% methanol, 192 mM glycine and 24.8 mM Tris. Phosphate-buffered saline (PBS) containing 5% non-fat milk was used to block the membranes for 2 h at room temperature. The membranes were incubated with anti-VEGF rabbit polyclonal antibody (Santa Cruz Biotechnology, 1:1000) and anti-β-actin mouse monoclonal antibody (Santa Cruz Biotechnology, 1:10,000) overnight at 4 °C. To wash off the redundant antibody, the membranes were repeatedly rinsed in TBS four times. Then, the membranes were incubated in peroxidase anti-rabbit IgG (Santa Cruz Biotechnology, 1:10,000) and peroxidase anti-mouse IgG (Santa Cruz Biotechnology, 1:10,000) for 1.5 h at 4 °C. After four washes in TBST, the membranes were imaged using a Bio-Rad ChemiDoc XRS+ by detecting enhanced chemiluminescence. Film autoradiograms were exposed from 5 to 15 min.

### Statistical analysis

The statistical analysis was performed with the SPSS 16.0 statistical package (SPSS Inc., Chicago, IL, USA). The data are expressed as the mean ± standard deviation values. The single factor analysis of variance (ANOVA) and *q* test were applied to evaluate five independent samples. Statistical significance was assumed at *p* < 0.05.

## Results

### Epidural scar adhesion

During the whole experimental period, none of the rats showed any signs of complications such as neurological deficits, disturbance of wound healing or wound infection.

In both the 30 mg/kg Sal B and 50 mg/kg Sal B groups, soft or weak fibrous adhesions were observed. However, in the 10 mg/kg Sal B and control groups, severe epidural adhesions were observed. Forced dissection of the epidural scar tissue would lead to serious bleeding and increase the risk of either nerve root injury or disruption of the dura mater. The grades of the epidural adhesions in the rats were determined based on the Rydell standards (Table 2).

**Table 2:** Rydell classification evaluation. The number of animals matching the various classification criteria was given

Group	Grade			
	0	1	2	3
Sal B (10 mg/kg, *n* = 10)	0	0	2	8
Sal B (30 mg/kg, *n* = 10)	6	3	1	0
Sal B (50 mg/kg, *n* = 10)	8	2	0	0
Control (saline, *n* = 10)	0	0	0	10

### Histological analysis

**Fig. 1:**
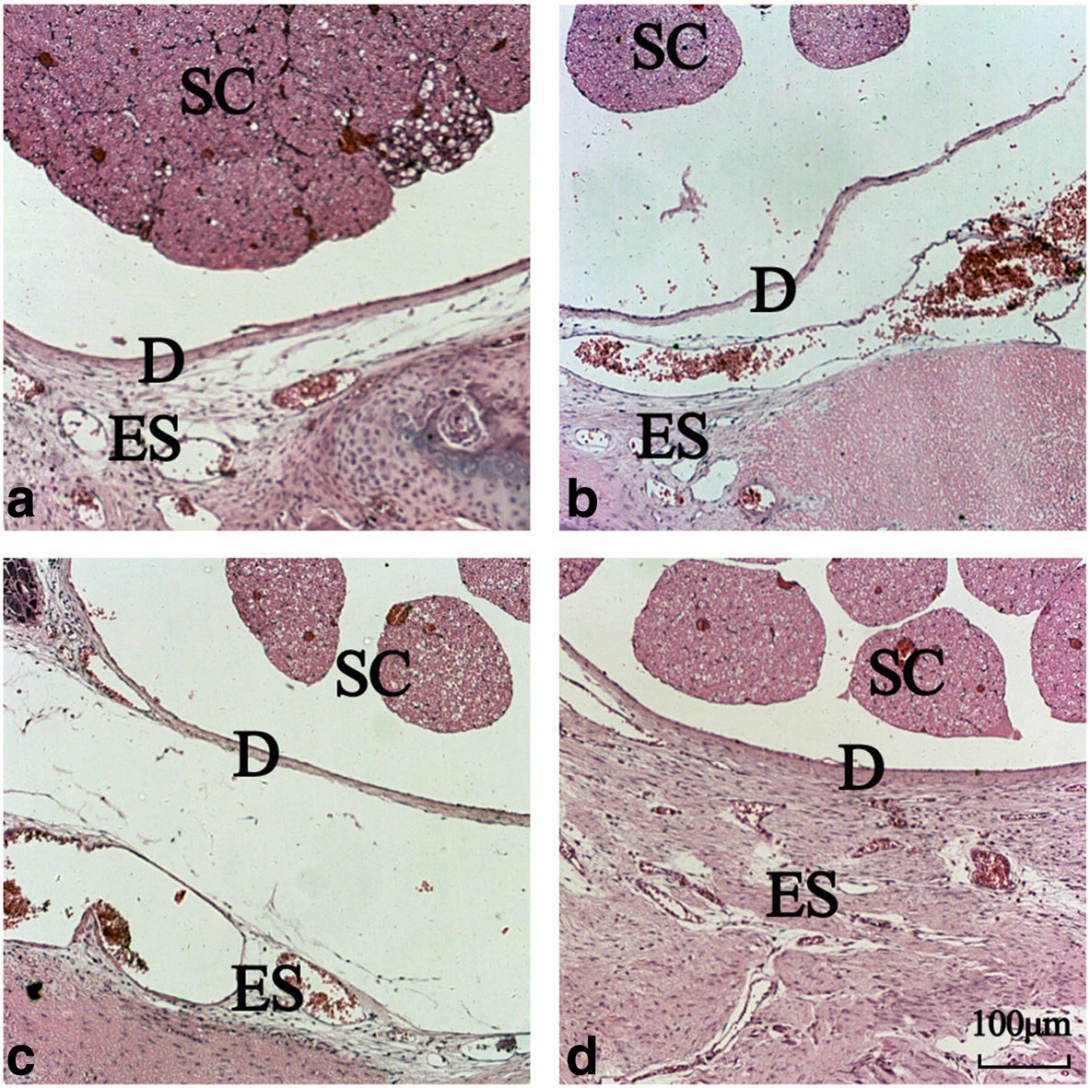
The status of epidural scar formation and epidural adhesion in the four groups. In the laminectomy sites of the 10 mg/kg Sal B and control groups, extensive adhesion and the pronounced scar tissues were observed (**a**, **d**). As to the laminectomy sites of the 30 mg/kg Sal B and 50 mg/kg Sal B groups, minimal adhesion and loose scar tissues were observed (**b**, **c**). *SC* spinal cord, *D* dura, *ES* epidural scar

The status of the epidural scar adhesions is further shown in Fig. 1. In the laminectomy sites of the 30 mg/kg Sal B and 50 mg/kg Sal B groups, minimal adhesion and loose scar tissues were observed (Fig. 1b, c). However, regarding the laminectomy sites of the 10 mg/kg Sal B and control groups, extensive adhesion and the pronounced scar tissues were observed (Fig. 1a, d).

### Effect of Sal B on fibroblasts proliferation

**Fig. 2:**
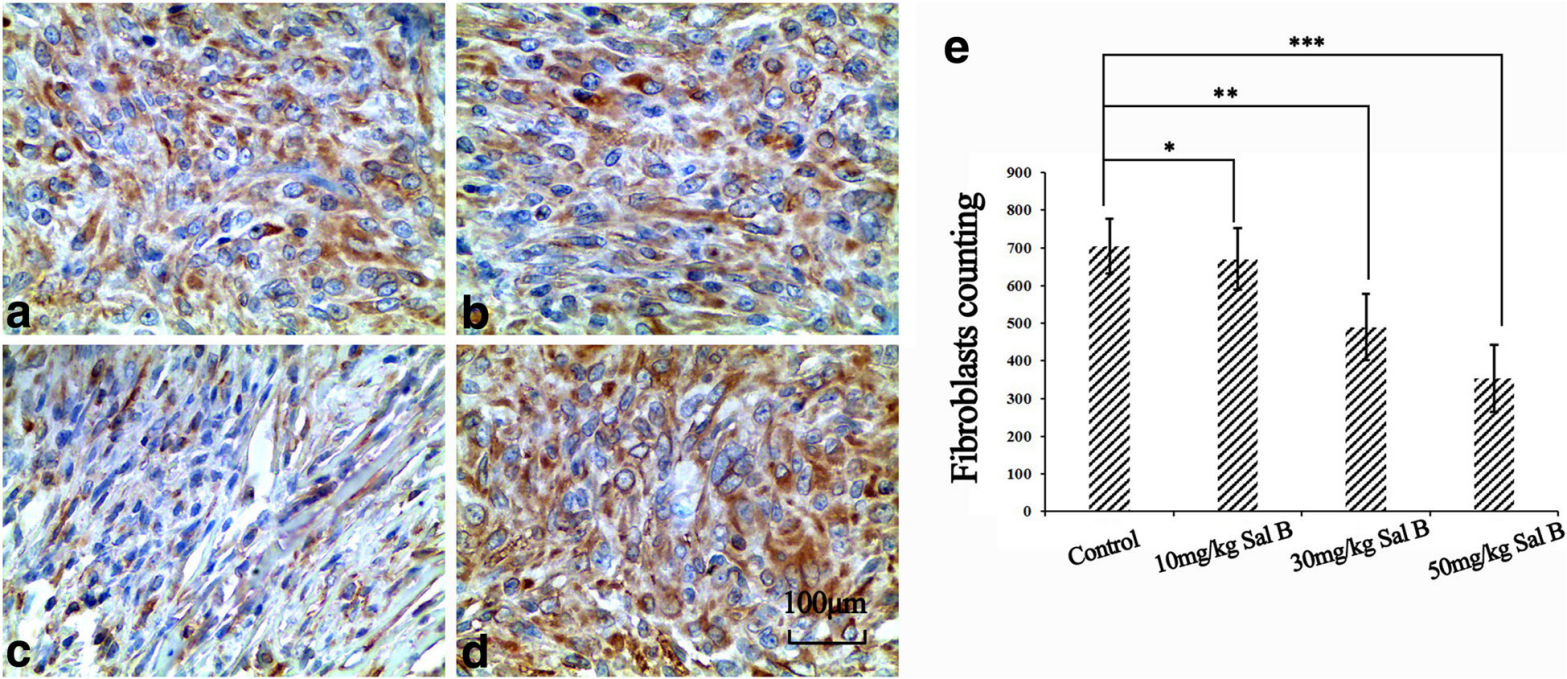
Detection of vimentin via immunohistochemistry and of the number of fibroblasts present in the epidural scar tissue from the four groups: 10 mg/kg Sal B group (**a**), 30 mg/kg Sal B group (**b**), 50 mg/kg Sal B group (**c**) and control group (**d**). Quantitative analysis was conducted based on positive cell counting, compared with the control group; all Sal B treating groups showed significant decrease (*p < 0.05 compared to the control group, **p < 0.05 compared to the control group, ***p < 0.05 compared to the control group) on fibroblasts proliferation (**e**). Values are presented as mean ± standard error of the mean

As shown in Fig. 2, the fibroblast counts suggested that compared with the control group (704.22 ± 72.38, Fig. 2d, e), both the 30 mg/kg Sal B group (489.77 ± 87.61, *p* < 0.05, Fig. 2b, e) and 50 mg/kg Sal B group (352.39 ± 89.35, *p* < 0.05, Fig. 2c, e) showed significant decrease in the number of fibroblasts at the wound site. At the same time, compared with the control group, the 10 mg/kg Sal B group (670.15 ± 81.29, *p* > 0.05, Fig. 2a, e) showed no significant change.

### Effect of Sal B on vascular regeneration

**Fig. 3:**
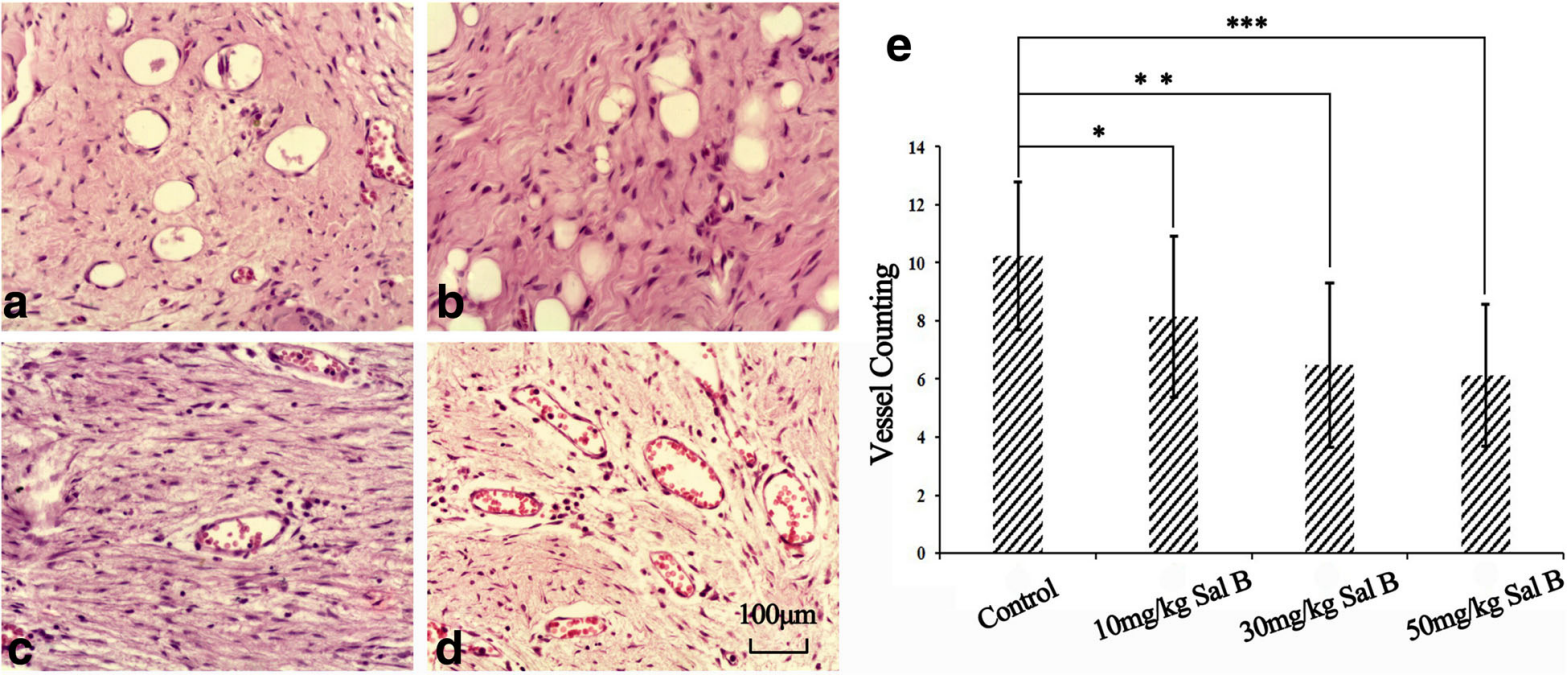
The status of the vasculature and the number of vessels identified in the epidural scar tissues from the four groups: 10 mg/kg Sal B group (**a**), 30 mg/kg Sal B group (**b**), 50 mg/kg Sal B group (**c**) and control group (**d**). Vessel counting quantitative analysis was conducted, compared with the control group; all Sal B treating groups showed significant decrease (*p < 0.05 compared to the control group, **p < 0.05 compared to the control group, ***p < 0.05 compared to the control group) on vessel formation (**e**). Values are presented as mean ± standard error of the mean

As shown in Fig. 3, the number of blood vessels in the epidural scar tissue suggested that compared with the control group (10.23 ± 2.55, Fig. 2d, e), all of the Sal B treatment groups showed significant differences as follows: 10 mg/kg Sal B group (8.15 ± 2.76, *p* < 0.05, Fig. 3a, e), 30 mg/kg Sal B group (6.48 ± 2.81, *p* < 0.05, Fig. 3b, e) and 50 mg/kg Sal B group (6.13 ± 2.45, *p* < 0.05, Fig. 3c, e).

**Fig. 4:**
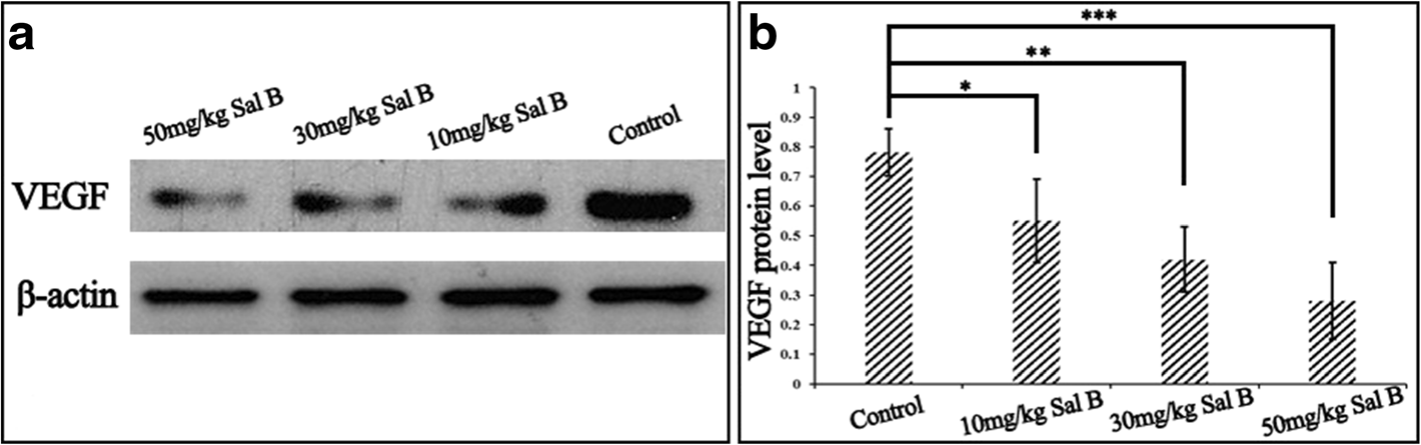
Effect of Sal B on VEGF expression in the epidural scar tissue. The expression levels of VEGF and β-actin in the epidural scar tissue from the four groups (**a**). The quantitative analysis was performed based on the greyscale value of straps. Compared with the control group, Sal B treating groups showed the significant decrease (*p < 0.05 compared to the control group, **p < 0.05 compared to the control group, ***p < 0.05 compared to the control group) on VEGF expression (**b**). Values are presented as mean ± standard error of the mean

In accordance with the aforementioned phenomenon observed based on H&E staining, Western blot targeting VEGF suggested the same trend as the H&E results. As shown in Fig. 4, compared with the control group, the expression levels of VEGF were significantly decreased in all of the Sal B treating groups. The effect of Sal B on inhibiting VEGF expressions showed the dose-dependent manner. Compared with other groups, 50 mg/kg Sal B had the most inhibiting effect on VEGF expression.

## Discussion

Globally, spinal surgery is annually performed in over one million patients [18]. EF is thought to be caused by spinal surgery and was first reported in 1948 [19]. It was reported that EF is the causative factor of pain in up to 36% of patients with failed back surgery syndrome [20]. Therefore, many researchers and surgeons have been working to overcome EF since it was first reported [6–10]. However, the underlying pathogenic mechanism of EF is still unclear, and EF is still one of most challenging problems after spinal surgery. One of the accepted treatment modalities for EF is the prevention or reduction of epidural scar formation [4, 21–23].

In previous studies, vascular regeneration was reported to be one of the most important mechanisms during wound healing [13, 24]. A series of growth factors and modulators were thought to be involved in regulating vascular regeneration. VEGF, as one of the critical factors of angiogenesis, has been reported to promote this process through regulating endothelial cell proliferation [13]. It was suggested that the neutralizing VEGF could reduce the proliferation of fibroblasts and decrease the regeneration of blood vessels, while the activating VEGF would result in the increased growth of fibroblasts and the neovasculature [25]. Thus, VEGF was selected as the main indicator in the present study. Our current results suggested the antagonistic effect of Sal B on the expression levels of VEGF in epidural scars. We hypothesized that one of the potential mechanisms of Sal B on curtailing EF was the antagonistic effect of Sal B on VEGF expression.

In our previous research, the results suggested the beneficial effects of Sal B on preventing EF with a dose of 30 mg/kg during the first four post-operative weeks. In the present study, we used three different doses for 8 weeks after the procedure to further evaluate Sal B’s safety and effectiveness. The present study suggested that a dose of 50 mg/kg Sal B resulted in the lowest levels of VEGF expression in the epidural scar and the lowest adhesion, and none of the animals died or showed any sign of complications until they were euthanized.

The latest studies in different fields have suggested the anti-fibrotic activity of Sal B in multiple organ systems such as pulmonary fibrosis, hepatic fibrosis and oral submucous fibrosis [14–17, 26–28]. These studies revealed the ability of Sal B to prevent fibrotic changes both in vitro and in vivo. It was reported that Sal B could prevent the fibrotic changes by inhibiting fibroblast proliferation, collagen deposition and alpha-smooth muscle actin expression [27].

In the present study, the post-operative intragastric administration of Sal B was employed. Compared with the intraperitoneal injection and local administration, intragastric administration has the advantages as follows: intragastric administration is able to keep Sal B blood concentration and attenuate the injury caused by the injection in a long-term setting and intragastric administration can accurately control the administration dosage. More studies looking into the optimal Sal B administration should be performed in future. At the same time, in the present study, no complications were observed. Though our results suggested the effects of Sal B on inhibiting the fibroblasts proliferation and blood vessel infiltration, no disturbance of wound healing or wound infection was observed. Considering the dose of 50 mg/kg is the highest concentration employed in the present research, more research should be performed to determine the optimal concentration for Sal B on treating EF in future.

## Conclusions

Post-operative intragastric administration of Sal B can prevent the formation of epidural scars. The effects of Sal B were dose-dependent, and a 50 mg/kg dose was suggested as the best effect in the present study. This study revealed that Sal B could be a potential therapy for EF and is worth perusing with further research.

## Abbreviations

EF: Epidural fibrosis
FBSS: Failed back surgery syndrome
Sal B: Salvianolic acid B
TGF-β: Transforming growth factor-β
VEGF: Vascular endothelial growth factor
